# Online Signals of Extremist Mobilization

**DOI:** 10.1177/01461672241266866

**Published:** 2024-07-31

**Authors:** Olivia Brown, Laura G. E. Smith, Brittany I. Davidson, Daniel Racek, Adam Joinson

**Affiliations:** 1 University of Bath, UK; 2 Ludwig-Maximilians-Universität München, Germany

**Keywords:** collective action, extremism, intergroup processes, violence

## Abstract

Psychological theories of mobilization tend to focus on explaining people’s motivations for action, rather than mobilization (“activation”) processes. To investigate the online behaviors associated with mobilization, we compared the online communications data of 26 people who subsequently mobilized to right-wing extremist action and 48 people who held similar extremist views but did not mobilize (*N* = 119,473 social media posts). In a three-part analysis, involving content analysis (Part 1), topic modeling (Part 2), and machine learning (Part 3), we showed that communicating ideological or hateful content was not related to mobilization, but rather mobilization was positively related to talking about violent action, operational planning, and logistics. Our findings imply that to explain mobilization to extremist action, rather than the motivations for action, theories of collective action should extend beyond how individuals express grievances and anger, to how they equip themselves with the “know-how” and capability to act.

The social psychology and terrorism literatures have historically diverged in their definitions and conceptualizations of extremist action. The social psychology literature provides a broad definition, encapsulating extremist action within the wider boundary of collective action, and as involving militant, illegal, and/or violent aspects that violate societal norms, and seeks to challenge or uphold the status quo, perpetrated on behalf of a radical or extremist group (Thomas et al., 2022). On the contrary, the terrorism literature has tended to focus specifically on terrorist action—defined as politically motivated violence, perpetrated by an individual and/or group that has undergone a process of radicalization (Ganor, 2002; Ruby, 2002; Sageman, 2017). This divergence in definitions can be traced to the sudden growth of terrorism research in the aftermath of 9/11, leading to its emergence as a distinct interdisciplinary field of study, with a predominant focus on terrorist acts and actors (Gordon, 2010; Silke, 2003, 2008; Youngman, 2020). And yet, it has been noted that progress within the terrorism literature has been stifled by a failure to integrate evidence and understanding from the social sciences, to comprehend the wider theoretical context in which we can understand how terrorism takes place (Gordon, 2010; Marsden, 2016; Wilkinson, 2006).

In response to this, we argue that if we can understand collective action as “any action that promotes the interests of one’s ingroup, or is conducted in politically solidarity” (Becker, 2012, p. 19), then it is possible to conceptualize extremist action (and terrorism) as a form of collective action, and importantly, to apply established social psychological frameworks of collective action to understand that action (Smith et al., 2020; Thomas et al., 2022). Indeed, the benefits to this have already been documented when examining how social psychological theory can inform understanding of radicalization processes (Smith et al., 2020, 2024).

That said, existing psychological frameworks of collective action have tended to focus on the underlying motivations for action (i.e., radicalization), rather than the mechanisms that might explain mobilization processes in terms of the “activation” of action. For example, the encapsulation model of social identity in collective action, or EMSICA (Thomas et al., 2009) and the social identity model of collective action (van Zomeren et al., 2004, 2018) emphasize the importance of perceiving collective injustice, sharing collective emotions such as anger, and identifying with the relevant ingroup to explain motivations for action. Thus, social psychology has arguably been more successful at explaining the motivational processes for collective action than the mobilization processes (i.e., the “tipping point”) for engaging in the action itself (Khalil, 2014; Livingstone, 2014). In parallel to this, the terrorism literature has been dominated by a focus on the radicalization processes that give rise to ideological beliefs that justify violence, as opposed to explaining the mechanisms through which individuals mobilize to violence itself (Moghaddam, 2005; Webber et al., 2020). For instance, Moghaddam’s (2005) staircase to terrorism model devotes more attention to outlining the attitudes, perceptions, and justifications of terrorist violence than it does to explaining how people might transition between radical opinion and radical action (McCauley & Moskalenko, 2017). Here, we argue that the radicalization process is not the same as the process of mobilization, by explicating how radicalization can be understood as the development of extremist ideologies and beliefs that can provide the motivation for terrorist action (Borum, 2011), whereas mobilization represents the process of engaging in such action (Klandermans & Oegema, 1987). We took advantage of the opportunity afforded by social media data, to conduct an exploratory study that examines how people’s interactions online prior to undertaking extremist action can provide insights into mobilization processes and indicators thereof.

## Explaining Motivations for Action Versus Mobilization to Action

The focus in terrorism and psychology research on the development of the motivation for action has been at the detriment of understanding and identifying the psychological states and behaviors that might indicate that a radicalized individual has decided to mobilize (i.e., to actually take action; Dudenhoefer et al., 2021; Horgan, 2012; Khalil, 2014; McCauley & Moskalenko, 2017; Stern, 2016). For example, while existing research enables us to understand why an individual might engage in collective action (Agostini & van Zomeren, 2021; Thomas et al., 2020) or how and why groups acquire ideologies that justify violence (Webber et al., 2020), little progress has been made in disentangling the mobilization process from the motivational process, such that it enables us to understand the differential predictors of the “activation” of action when compared with the predictors of motivation for action (e.g., van Zomeren, 2013). Indeed, many studies of collective action, for example, measure participants’ intention to act and do not measure behaviors or empirically examine the mobilization process itself (i.e., how people move from intention to action), which runs the risk of conflating motivation with activation (Klavina & van Zomeren, 2020; Lantos et al., 2020).

In turn, this has led to an inability to distinguish between the beliefs and motivations that justify and or/support an extremist action, and the knowledge, activities, and behaviors that enable the actors to engage in that action (Borum, 2011; Khalil, 2014). As highlighted by Horgan (2012), “A lot more people are radicalized than will ever become involved in terrorism. . . . Rooting out radicalization has become a proxy for pre-empting terrorism.” This means that theories of collective action and radicalization cannot usefully explain which people within an extremist group will mobilize to take extremist action. This is inherently linked to the “specificity problem” (Sageman, 2004)—the challenge of being specific in predicting who will perpetrate an extremist act.

We propose that to help solve the specificity problem and better explain mobilization, theories of collective action need to incorporate an understanding of how people equip themselves with “know-how” and the capability to act (Beck, 2008; Boyns & Ballard, 2004). Since the 2000s, the move toward social psychological explanations of mobilization that focus on collectivized feelings of injustice, anger, and efficacy, and shared social identification has meant that the role of resources, the development of the capability to act, and operational behaviors has been somewhat underplayed (Beck, 2008). Yet, both are necessary. As argued by resource mobilization theory (Ferree & Miller, 1985; McCarthy & Zald, 1977), while grievances are a necessary condition for mobilization, they are not sufficient for individuals to act. An individual (seen as a rational actor) requires the resources and know-how to undertake a violent act (Boyns & Ballard, 2004). Similarly, the theory of planned behavior (Ajzen, 1991) describes how an individual’s beliefs about perceived behavioral control need to be combined with attitudes and norms for a behavior to occur (i.e., for them to mobilize). This does not imply that collectivized grievances, hate, anger, and ideological beliefs are unimportant, but rather that individuals are unlikely to act without accompanying know-how and resources that would enable them to act (Beck, 2008).

## The Role of Online Interactions in the Mobilization to Action

To investigate the issues above, we can look at people’s social media interactions prior to their mobilization. Social media interactions provide a cloudy window into people’s behaviors and motivations prior to, and during, collective action (Smith et al., 2023). However, the role of social media interactions in mobilization remains undertheorized and underexplained in comparison with its role in radicalization (Smith et al., 2023; Stern, 2016).

Evidence suggests that people radicalize and polarize online (that is, develop and intensify their commitment to groups and those groups’ ideas) through validating interactions about grievances (Smith et al., 2020, 2023). Indeed, the rise in extremist^1^ content online has caused an increasing concern that the internet can serve as a vector for radicalization. Over a million accounts were suspended on Twitter between 2015 and 2017 for promoting terrorism, and over 150,000 YouTube videos were removed for promoting violent extremism (Allcott et al., 2019; Allcott & Gentzkow, 2017). The global proliferation of extremist content online has compounded the challenges governments and organizations face in determining which individuals (and what content) might be a risk to public safety (Macdonald et al., 2019; Scrivens, 2021). In response, state and private-sector resources have been directed toward this task, including monitoring social media platforms to identify the digital traces that are associated with increased risk of extremist action (Home Office, 2018; Macdonald et al., 2019).

However, the increase in high-profile terrorist attacks in which online communications have been implicated suggests that online radicalization and polarization do not provide useful and generalizable signal of mobilization that can be used to prevent attacks (Scrivens, 2021; Macdonald et al., 2019; Quiggin, 2017). One of the reasons for this is that the frequency of extremist actions offline is low in comparison with the vast volume of extremist content online, with 5,226 terrorism-related incidents reported in 2021 (National Consortium for the Study of Terrorism and Responses to Terrorism [START], 2022). Although many people express ideologically extreme and hateful views, “very few follow up with violent actions” (Sageman, 2011, p. 117). The sheer volume of extremist material obfuscates the signal of mobilization. More significantly, the difference between the volume of extremist content online and the number of incidents offline suggests that extremist content per se cannot provide a sufficiently granular or precise signal of mobilization, or insight into mobilization. Thus, trying to understand the mobilization of extremist action by investigating radicalization processes, or extremist online content, is unlikely to be appropriate. And yet, the examples above highlight the potential of using online data to investigate the mobilization process, and generate questions as to how and why the use of social media relates to the mobilization of violent extremism (rather than radicalization), and, in turn, how social media data might be used to explain that mobilization (Conway, 2017; Munk, 2017).

## Digital Signals of Mobilization

One phenomenon that can be leveraged to investigate how social media data might be used to investigate mobilization is *leakage* of intentions during online interactions. Leakage was originally defined as when an individual “intentionally or unintentionally reveals clues to feelings, thoughts, fantasies, attitudes, or intentions that may signal an impending violent act” and has later been referred to as “the communication to a third party of an intent to do harm to a target” (Meloy & O’Toole, 2011, p. 1; O’Toole, 2000, p. 1).

When perpetrators of extremist action interact online, they may “leak” cues that provide a potential signal (or warning sign) that they are prepared (or preparing) to mobilize (Meloy & O’Toole, 2011; Rose & Morrison, 2023). This leakage can be understood as a behavior enacted by an individual and can reflect a diversity of actions and behaviors. For instance, leakage could include an individual telling others about their plans to mobilize, gathering or sharing information about how to do so, demonstrating their capability through sharing information on weapons, or expressing violent intent (Brynielsson et al., 2013; Dudenhoefer et al., 2021; Gill, 2015). Leakage behaviors, therefore, may provide a signal that an individual is likely to mobilize. This may be because, (for example) if people are using online interactions to equip themselves and others with know-how, this may increase their operational capability. Crucially, leakage leaves *digital traces*—“records of activities and events that are produced, stored, and retrieved using information technologies,” that can include text from social media posts and paralinguistic cues such as punctuation and word count (Vial, 2019, p. 1).

In this research, we explore whether digital traces of leakage behaviors are likely to be included in the content of online posts of individuals who have subsequently mobilized to extremist action (e.g., seeking information, talking about the logistics, sharing grievances; Pennebaker, 2011; Rose & Morrison, 2023; Schuurman et al., 2018). In addition, we explore whether there are other paralinguistic features and metadata (e.g., frequency and length of posts) that could be used to distinguish between extremists who intend to mobilize and those who do not (Scrivens et al., 2023). For example, punctuation such as exclamation points can signal intensity or emotive force (Quirk et al., 1985; Rubin & Greene, 1992), or that the preceding sentence was an “exclamation or strong assertion” (McArthur et al., 2018). Because posting publicly online requires people to have made a conscious decision to broadcast their opinions, this behavior is likely to leak (at least partly) their internal motivations and attitudes. Therefore, taken together, the evidence suggests that digital trace data (e.g., content of posts, punctuation) have the potential to inform our understanding of how individuals behave online when they transition from consumers/creators of extremist content toward mobilization to extremist action.

## The Present Study

The potential that digital trace data provide to investigate mobilization has been limited by a lack of studies that include an appropriate comparison between the online communications data of radicalized individuals who have and have not mobilized (Holt et al., 2019; Khalil, 2014; Zannettou et al., 2020). We address this by presenting a unique database of social media posts, authored by a sample of extremists who had been convicted for terrorism-related offenses and a sample of those who had not. By leveraging the idea of leakage (O’Toole, 2000) and theories of mobilization (Smith et al., 2023; Tausch et al., 2011; van Zomeren et al., 2008, 2018), we aimed to identify the digital trace variables that could be used to explain the differences in online behavior of people who mobilized to extremist action versus those who were radicalized (i.e., held similar extremist views but did not take action). Doing so enabled us to develop theoretical propositions that explain how individuals behave online once they have moved “up” the radicalization “staircase” (Moghaddam, 2005), and passed the psychological tipping point for engaging in extremist action. Thus, our research aimed to contribute toward developing theoretical propositions to help identify the “needle in the haystack” and extend the possibilities of using digital trace data to prevent terrorist attacks (Macdonald et al., 2019; Quiggin, 2017; Sageman, 2011; Scrivens, 2021; Scrivens et al., 2019).

To address our study aims, we first created a database containing 119,473 posts, authored by 26 users convicted of a terrorism-related offense and 48 users who had not been convicted. To investigate whether digital trace data can be used to inform our understanding of users’ online behavior when they intended to mobilize to extremist action (and thus inform theories of mobilization), we conducted three sets of analyses. We had no hypotheses in our analyses due to the exploratory nature of our research. In Part 1, we conducted an exploratory content analysis on a subsample of posts to contextualize and deepen our understanding of our data set and inform our quantitative analyses in Part 2 and 3. Next in Part 2, we utilized the findings from Part 1 to inform our topic modeling approach, with the aim of identifying themes and patterns across our entire data set that could then be used as variables in our modeling in Part 3. In Part 3, we combined the results of our prior analyses to inform the development and testing of random forest models that predicted the probability that posts were authored by a person who had been convicted of mobilizing to extremist action versus those who had not. This then provided a digital signal of extremist mobilization, indicating the differences in online communication behavior between people who were radicalized and intending to mobilize to extremist action, and those who were radicalized but not intending to mobilize.

## Database

Ethical approval was obtained from the University of Bath (ref. 20-107). To create our database, we focused specifically on right-wing extremists.^2^ There have been a number of high-profile right-wing terrorist attacks in recent years that have illustrated the difficulty of identifying signals of mobilization in digital data and the consequences of not doing so. For example, the individual charged with killing 11 people at a synagogue in the United States demonstrated increasingly hateful and anti-Semitic views online in the weeks preceding his offense, before announcing his intentions on *Gab* in the moments before perpetrating his attack (McIlroy-Young & Anderson, 2019). The perpetrator of the Christchurch terrorist attack in New Zealand shared his plans on Twitter and 4Chan in the days and hours preceding the attack, before livestreaming his actions on Facebook (Macklin, 2019). While these incidents are indicative of the challenges associated with efforts to monitor online material, they also highlight the potential of using data from the online communications of terror actors to examine online signals of mobilization and further understand this process (Scrivens et al., 2019). This is especially pertinent in the case of right-wing terrorist attacks, in which individuals tend to act alone, using the internet in their preparation and planning, but rarely display the offline behaviors that have historically been indicative of future violence (e.g., coordinated planning across group members, reconnaissance activities; Gill, 2015).

To build a database of convicted right-wing extremists, we drew on open-source intelligence (OSINT; for example, news reports, court reports) and existing terrorism databases (Ravndal, 2016; START, 2022). Initially, we had intended to only include individuals who were convicted of terrorism offenses. However, until 2021, U.S. terrorism laws were focused predominantly on foreign actors (The White House, Washington, 2021); thus, excluding individuals convicted of cases of racially motivated violence and/or hate crimes would have reduced our sample and failed to capture the prevalence of right-wing extremist actions between 2015 and 2021 (Blackbourn et al., 2019). Therefore, and to ensure we captured the broadest forms of extremist actions that were consistent with our definition of action taken in the interests of one’s ingroup, or in politically solidarity (Becker, 2012), our inclusion criteria for our database were as follows: (a) we only included individuals for whom there was a clear reference to their offense being motivated by right-wing extremist beliefs; (b) we only included individuals from English-speaking countries so that any data could be analyzed without the need to use translation software; and (c) we only included individuals convicted of offenses between 2015 and 2021 to increase the chances that offenders were active users of social networking platforms (Holt et al., 2019). We identified 139 individuals who met our inclusion criteria, and these individuals are hereafter referred to as the “mobilized” sample. Within this sample, the majority of offenders were convicted in the United States (75%), with 20% convicted in the United Kingdom and the remaining 5% in Australia, Canada, and New Zealand.

Next, using OSINT, we identified that 135 individuals in our database were active users of at least one social networking platform (e.g., Facebook, Telegram, Twitter). We opted to focus on three specific platforms for our later analyses—Iron March, Gab, and Discord (see Table 1). These were the three platforms most commonly used by the individuals in our database (*n* = 45) and data from these platforms were available in publicly accessible repositories. We used publicly accessible data sources to adhere to the British Psychological Society (BPS) Ethical Guidelines for Internet Mediated Research. Because we used data from existing public repositories, no primary data collection took place for the purposes of this research. There are existing examples within the literature in which data from these three repositories have been utilized to study far-right communities (Kennedy et al., 2022; McIlroy-Young & Anderson, 2019; Munn, 2019; Scrivens, 2021; Zannettou et al., 2018). Furthermore, and in accordance with BPS guidance, we have ensured that our presentation of the data protects the anonymity and privacy of users, and the data are stored in adherence with the General Data Protection Regulation (GDPR) 2018.

**Table 1.** Summary of Database (N = 74 Users, n = 200,211 Posts).

Site	Source of data	No. of users	Total no. of posts	No. convicted user posts (%)
Iron March	Data deposited online in November 2019. Data were downloaded from https://www.bellingcat.com/resources/how-tos/2019/11/06/massive-white-supremacist-message-board-leak-how-to-access-and-interpret-the-data/	11 convicted 9 nonconvicted	7,921	7,219 (91.1%)
Discord	Data sourced from Unicorn Riot (independent media collective) https://discordleaks.unicornriot.ninja	14 convicted 43 nonconvicted	112,541	11,524 (10.2%)
Gab	Data sourced from https://files.pushshift.io/gab/	9 convicted 18 nonconvicted	79,749	20,794 (26.1%)

Next, we searched the Gab, Iron March, and Discord data repositories for the online posts of the individuals in our mobilized sample of people convicted of a terrorism-related offense. People with extremist views often adopt “digital aliases” (i.e., usernames) on social media platforms to hide their real identity (Brynielsson et al., 2013) and thus we needed to correctly identify the usernames individuals had adopted online. Two coders independently implemented a coding framework to ensure we correctly matched individuals to their digital aliases (see Supplementary Table 1). Of the 26 individuals that we were able to identify from their online usernames, three were convicted of a violent offense involving the murder of one or more people, seven of violent assault, two of plotting an act of terrorism, seven of possession of terrorism-related materials or weapons, three of membership of a terrorist organization, three of making violent threats, and three of distributing harmful content.

To generate a comparison data set of individuals who held right-wing extremist beliefs (i.e., were radicalized) but had not mobilized to extremist action, we conducted a further OSINT search, drawing on reports from nonprofit organizations that exist to counter hate online (e.g., Hope Not Hate, Anti-Defamation League). We identified a sample of 48 individuals^3^ who met the following criteria: (a) they had not been convicted of a terrorism-related offense; (b) they could be readily and reliably identified as a right-wing extremist (see Supplementary Table 2); and (c) they were known users of Iron March, Gab, or Discord. By applying these inclusion criteria, we created a sample of individuals who had not mobilized, meaning that we were able to use expression of support for extremist action as a baseline with which to compare the online behavior of those who had mobilized. We applied the same coding framework as we did for mobilized users to ensure individuals were correctly matched to their digital aliases (Supplementary Table 1).

Our final database consisted of 200,211 posts authored by 27 individuals who had mobilized and 48 who had not. All posts in our database were dated between 2011 and 2019. There was a high degree of variance in the length of time that individual users’ posts spanned (1 month–8 years) and a large range of posts across users (112–34,345). This meant that there was variance within the convicted sample in terms of the time lag between their final posts and when they mobilized.

All code and transformed data are available on the following OSF repository: https://osf.io/6dxep/?view_only=685bfa463937485cb559baca69398c0e. The raw data are not available due to the nature of the content and to ensure that we adhere to the terms of service for each of the social networking platforms; however, we note that readers may access the public repositories from which these data were obtained (see Table 1). The methods and scripts used to transform the raw data for the topic modeling and machine learning approaches are available allowing the initial approach to be understood transparently and openly. The quantitative data were analyzed using R, Version 4.1.0 for macOS (R Core Team, 2020). The specific R packages for each analysis are detailed in the “Method” and “Results” sections for each Part. The qualitative data were analyzed in Nvivo (for the content analysis) and SPSS (for the chi-square comparisons of coded categories). The designs and analyses were not preregistered as we had no hypotheses. In each section of our analyses, we report how we determined our sample size, all data exclusions (if any), all manipulations, and all measures in the study.

## Part 1: Content Analysis

We first conducted a qualitative content analysis to explore differences in content between the two groups (mobilized and not mobilized) and to begin developing an understanding of what type of content might be used to identify a signal of mobilization to action. The content analysis was also essential to guide our quantitative analysis in Part 2 and 3.

Despite the rise of computational methods in psychological science, we concur with Mills’s (2018, p. 600) assertion that the unique value of qualitative methods remains, as “we cannot understand human behavior and social action, without contextualizing the data and understanding the environment in which the data about social action occurs.” We further contend that qualitative analysis is especially important in this context as extremist forums are characterized by specific styles of language and vocabulary not used by the general population (Cohen et al., 2014; Kosse, 2022; Peeters et al., 2021). As such, relying solely on a computational approach would be unlikely to succeed, given the depth of knowledge required to understand the meaning and context of the text (Cohen et al., 2014). Part 1 of our analysis was, therefore, necessary to provide a descriptive overview of content posted within extreme right-wing online communities and to inform both the methodological choices and the interpretations of our quantitative analyses in Part 2 and Part 3. This approach to has been referred to as theoretically informed data science, in which predictions about human behavior (in our case mobilization to extremist action) and the algorithms designed to make those predictions are informed by psychological theory and contextual understanding (Hinds et al., 2022).

### Method

#### Sample

We identified a subsample of 10 individuals who had mobilized and 10 individuals who had not mobilized. We employed a matched sample technique in that all users were comparable in terms of their influence (e.g., group leaders) and only differed in terms of whether they had mobilized to extremist action. This was to increase uniformity across the groups and make our comparisons by mobilization more valid. The 20 users identified for our analysis had authored a total of 36,289 posts in the database. Of these posts, 13,237 posts were from mobilized users and 23,052 were from nonmobilized users. Prior to the analysis, we removed all noncodable posts (*n* = 3,504), including blank posts and posts that only contained images (as our analysis was focused on the textual content of posts). Once noncodable posts had been removed, there were 32,785 posts remaining, with 20,303 posts from nonmobilized users and 12,306 posts from mobilized users. The number of posts per user ranged from 568 to 5,103 for individuals who had not mobilized and 112 to 3,668 for users who had mobilized.

#### Analytic Strategy

We conducted a content analysis on the 32,785 posts, keeping the mobilization status of the individuals blind to the coder (see Supplementary Table 3). Content analysis allows samples of text to be compared (in this case, the posts of individuals who had mobilized and individuals who had not) by grouping text into meaningful categories (Weber, 1990). We chose content analysis as the data can be analyzed descriptively (e.g., using quotes to provide context to the material) and quantitatively by comparing scores of latent constructs derived from the coding process (e.g., by comparing scores in higher-order categories across the mobilized and had not mobilized groups; Hsieh & Shannon, 2005). We adopted an inductive approach, with codes derived from the data, rather than drawing on preexisting theory (Kondracki et al., 2002).

Following the steps outlined by Hsieh and Shannon (2005), the first author began by familiarizing themselves with the text and annotating the data to identify initial codes. Once all of the data had been coded and initial codes identified, higher-order categories were derived from the initial codes, based on how the codes were related to one another. Next, to check for reliability, the first author and a second coder re-coded a sample of 2,000 posts (with an equal number of posts from each user). A high level of agreement was obtained, *k* = .94 (Everitt, 1996). To protect the anonymity of users, no usernames or identifying information were included in the quotations used to illustrate the results (British Psychological Society, 2021). To compare the posts of users who had mobilized and users who had not, we then conducted a chi-square test of independence on the raw counts of each higher-order category. Sufficient power was obtained for all chi-square tests (Faul et al., 2007).

### Results

We identified 23 codes that were then grouped into nine higher-order categories (see Supplementary Table 3 for the coding dictionary). For example, both “connecting with others” and “recruitment” were collapsed into the higher-order category of “Group Formation,” and both “sharing literature” and “sharing news” were included in the higher-order category of “Sharing Information.” We then conducted a comparison of the nine higher-order categories across the posts of mobilized and nonmobilized users (Table 2). Mobilized users’ posts contained significantly more content in all the coded categories apart from “Intragroup Debate” and “Inciting Violence,” for which there was no difference between the two groups.

**Table 2.** Comparison of Higher-Order Content Categories in Mobilized Users Sample (n Posts = 12,306, N Users = 10) and Users Who Had Not Mobilized Sample (n Posts = 20,479, N Users = 10).

Higher-order category	Definition	Mobilized	Had not mobilized	χ^2^	*p*
Group Formation	Forming a group and connecting other forum users together.	2.94%	1.29%	112.243	<.001
Hateful Content	Hateful and derogatory content in relation to extreme right-wing beliefs—racism, homophobia, misogynism etc.	3.71%	2.38%	47.224	<.001
Information Sharing	Information being shared to other members of the online forum	5.88%	4.34%	38.817	<.001
Intragroup Debate	Debate amongst members of the forum, including ideological debate, historical debate and debate around group norms	7.13%	7.12%	0.007	0.930
Operational Security	Advice on how to operate effectively—e.g., what to wear, what security to incorporate onto their technological devices	1.56%	1.10%	13.038	<.001
Threat of Violence	Content that reflects an individual threatening to engage in act of violence	0.30%	0.11%	14.932	<.001
Offline Action	Reference to action offline—flyering, postering, lobbying etc	0.81%	0.28%	43.818	<.001
Weapons	Mention of weapons	0.77%	0.06%	117.521	<.001
Inciting Violence	Content that incites other people to engage in acts of violence	0.28%	0.32%	0.648	.421

That we identified significantly more content in the mobilized group in relation to Operational Security, Threats of Violence, as well as references to Weapons and Offline Action provides support for the suggestion that evidence of operational capability and know-how can explain why certain individuals mobilized to action and others do not (see Beck, 2008). Across these higher-order coded categories, the content was indicative of individuals equipping themselves with know-how and resource (e.g., in content relating to Weapons, “our ammo has arrived” and content relating to Operational Security, “Before doing something having a scout of the area, know how you’re going to get there, how you’re going to leave”), as well as indicating a degree of capability required to initiate action (e.g., in content relating to Offline Action, “I’ve already got a group of nationalists training in boxing/kickboxing” and Operational Security, “just carrying damn near any electronic device on your person means your location is potentially compromised”).

Interestingly, we found higher instances of Group Formation and Information Sharing in mobilized users’ posts. This suggests that individuals who mobilize are not only likely to use the internet to develop know-how and capability for their own future action, but that they are actively engaged in recruiting (e.g., in the case of Group Formation, “are you interested in joining? We can meet next weekend”) and sharing information and resources with others (e.g., in content relating to Information Sharing, “Read [redacted] first though”), to facilitate their engagement with action. These roles and functions within online communities have tended to be treated as separated, with an assumption that individuals inhabit one or the other (Phadke & Mitra, 2021). In contrast, our findings suggest that individuals who mobilize not only develop their own capability but that they proactively engage in strategies to enhance the overarching capability of the group (i.e., through recruiting additional members and sharing resources). Future research might examine this in more detail by tracking role transitions in online communities over time (Davidson et al., 2019).

Furthermore, we found significantly more Hateful Content in the mobilized users’ posts. While this is suggestive that hateful language might be more commonly adopted by individuals who have the propensity to mobilize, the vast evidence base that demonstrates the regularity of hateful content across far-right online communities (e.g., Scrivens, 2021; Zannettou et al., 2018) and suggests that it may not be the most reliable indicator of mobilization. Indeed, consistent with collective action theory, we know that individuals in support of action and who identify with a specific group will collectivize grievances and consensualize around expressions of anger and emotion (i.e., Hateful Content: “I hate Jews so, so much”), but that these motivations for action are not necessarily indicative of who might mobilize to action (see also Smith et al., 2023).

Finally, that we found no significant difference in Intragroup Debate and Inciting Violence might be explained by the commonality (or lack of) with which these higher-order categories were coded. Intragroup Debate was the most commonly coded category and appeared in 14.25% of all posts, whereas Inciting Violence was the least commonly coded category, appearing in 0.5% of posts. That Intragroup Debate this was commonly coded for is consistent with prior research which has found that online communities provide a platform to share and express opinions, alongside an opportunity for ideological learning (De Koster & Houtman, 2008; Hardy, 2023; Lee & Knott, 2022).

### Discussion

Part 1 of our analysis demonstrated differences in the content of posts authored by individuals who mobilized and those who did not, and, importantly, suggests that these differences relate to content about operational capability and know-how in those that mobilize. Therefore, in Part 2, we conducted a second analysis on our entire sample of posts, aiming to investigate how the coded categories identified in Part 1 mapped at scale, and how these categories could be used in the machine learning modeling in Part 3 that will test our primary empirical aim—to investigate whether digital traces can be used to identify signals of mobilization to extremist action.

## Part 2: Topic Modeling

Building on Part 1, Part 2 of our analyses sought to identify themes and patterns within the entire data set that could then be used as variables (or features as they are termed in computational methods) in Part 3 of our analysis—the machine learning modeling.

Drawing on recent advances in computational grounded theory, we used the higher-order categories identified in the qualitative analysis to inform our topic modeling and investigate how these categories mapped at scale across the entire data set (Jaworska & Nanda, 2018; Nelson, 2020; Storer & Rodriguez, 2020). By explaining our topic modeling processes in detail here, with reference to the qualitative findings in Part 1, we aim to overcome some of the criticisms of computational approaches as being a “black box process” (Pasquale, 2015)—that while it is possible to observe the inputs and outputs of a model, it is not always possible to ascertain how each relates to the other, creating a lack of generalizability and interpretation of models. Thus, Part 2 of analysis aimed to (a) extend our descriptive analyses conducted in Part 1 by modeling the entirety of our sample and (b) identify topics within the data that could then be used in Part 3 of our analysis.

### Method

#### Sample

For our topic modeling sample, we first cleaned and processed the full database of posts (*n* = 200,211). At this stage, one user was removed. This was because this user had a high number of posts (*n* = 34,345), accounting for almost a sixth of the entire sample, and we were concerned that this would skew our analysis. In addition, any posts that contained less than four words were removed so that we had sufficient numbers of words per post for topic modeling. This left us with a final sample of 119,473 posts for our topic modeling analyses (86,854 from nonmobilized users and 32,619 from mobilized users).

#### Analytic Strategy

Topic modeling is an unsupervised approach, where the underlying model identifies patterns in data and organizes them into topics . The topic modeling analysis was conducted in *R* using the *text2vec* package (Nikolenko et al., 2017). First, we tokenized the text and removed all stop words using a custom list for our specific data set (see https://osf.io/6dxep/?view_only=685bfa463937485cb559baca69398c0e). This produced a vocabulary and document-term matrix that could be used to perform topic modeling. *text2vec* uses the Latent Dirichlet Allocation (LDA) DA algorithm and we internally set lambda at 0.4 to balance the relevance of words in topics alongside the probability of their occurrence (Selivanov, 2022).

We did not adopt a more contextualized approach, such as BERTopic or Word2Vec for several reasons. The first was that our LDA approach has clear, well-established metrics with which to establish topic coherence and has been widely used across social science research (Asmussen & Møller, 2019). LDA is also suited for relatively small data sets and did not require the training of an embedding model beforehand, unlike approaches such as Word2Vec, for which we would not have had sufficient training data (Egger & Yu, 2022). Due to the sensitive nature of our research context, we did not deem it appropriate to use pretrained models and/or embeddings that were not specific to our data set and did not want to take the risk that any effect identified were artifacts of these pretrained models. Finally, as LDA directly employs words to fit topic clusters and does not make use of any pretrained embeddings, its outputs are fully transparent (Blei et al., 2003). This was important as it enabled us to compare the outputs with the findings of our qualitative analyses in Part 1.

**Figure 1.**
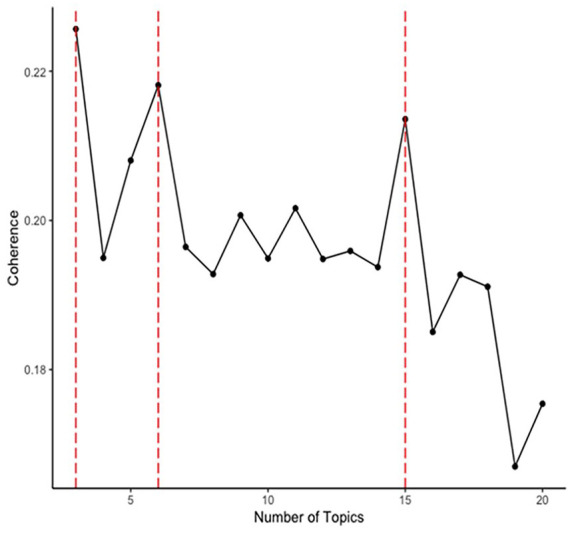
Statistical Coherence Plot for Topic Models.

Informed by our findings from Part 1, we iterated through topic models with varying numbers of topics (*k* = 3:20), which means the algorithm was clustering the words into three to 20 topics. This was then used to identify what number of *k* topics best aligned with the results of the content analysis. To do this, we extracted the 50 words most frequent terms per topic and examined their conceptual overlap with the qualitative content analysis findings to identify the most conceptually coherent numbers of topics. We then compared the utility of this mixed-methods approach with purely data-driven approaches for finding the most appropriate number of topics (in relation to our primary empirical aim: identifying the pattern of differences between the online behavior of mobilized versus nonmobilized users). For the data-driven approach, we calculated the statistical coherence per topic model, using the average cosine similarity on the normalized pointwise mutual information values (see Figure 1; Röder et al., 2015). This strategy meant that in Part 3 we were able to compare the validity of a classifier that was informed by the qualitative analysis against the validity of purely data-driven classifiers.

### Results

To aid in the process of iterating through topic models to identify which aligned best with the results of the content analysis, we assigned the topics within the models a representative label, based on the terms included in the topic. After iterating through topic models with between three and 20 topics, we found that the four-topic model was most aligned with the results of our content analysis in Part 1 (see Table 3). The four-topic model captured all nine higher-order categories identified in the content analysis and the terms within each of the topics were qualitatively similar and could be identified within coded sentences of our qualitative analysis. For example, the qualitative higher-order category of Hateful Content included “Death to kikes. Death to fags. Death to non-whites” and several terms within this quote are represented in the topic of Hate Speech (see Table 3). In addition, the number of coded occurrences of each of the categories was also reflected in the way in which they were distributed in the topic models. For example, Topic 4 aligned with one category (Intragroup Debate), corresponding with the fact that this was the most frequently qualitatively coded category, whereas Topic 3 (Violent Action) was represented by five categories (Operational Security, Threat of Violence, Offline Action, Weapons, and Inciting Violence) which were the least frequent of the coded categories.

**Table 3.** Overlap of Four-Topic Model With Higher-Order Categories From Content Analysis (Mobilized Sample, n Posts = 32,619, N Users = 26; Users Who Had Not Mobilized Sample, n Posts = 86,854, N Users = 48).

Topic	Description of topic	Example terms in topic	Content analysis category	Example quotes from qualitative analysis
Topic 1 Online Communication	Terms that relate to sharing communicating online (e.g., terms such as gab, twitter, discord) and sharing information (e.g., terms such as link, video, book).	Post, read, gab, server, everyone, account, link, site, siege, chat, stormer, discord, email, channel, week, check, video, twitter, pm, message, contact, book	Information Sharing	“Read [redacted] first though”
			Group Formation	“Deleted some messages in my inbox. PM me with your guy’s skype and i will personally do the interview myself tomorrow.”
Topic 2 Hate Speech	Terms that are associated with hate speech within far-right communities (e.g., jew, ni*ger, k*ke).	Jew, white, fuck, people, k*ke, nig*er, racist, women, jewish, hate, cuck, retard, Israel, trump, lie, faggot, stupid, nazi, genocide, kill, racism, bullshit	Hateful Content	“6 word speech. Gas the k*kes. Race war now”
Topic 3 Violent Action	Terms that relate to violent activities (e.g., shoot, punch, terrorist, attack, gun), and reference law enforcement (e.g., cop, arrest, police).	Antifa, Charlottesville, gun, terrorist, attack, cvill, wear, rally, violent, cop, arrest, police, shoot, mask, punch, defense, shirt, thug, torch, protest, gang	Operational Security	“no one will commit any crimes so no one will get arrested, isn’t that right FBI?”
			Threat of Violence	“I wonder if people realize this shit will consist of real violence and it is going to accelerate a lot more before we can achieve our our goals”
			Offline Action	“I’m at a protest with antifa”
			Weapons	“One of our major problems at the moment is getting money for more guns and more ammo and more geat”
			Inciting Violence	“This isn’t a political struggle. This is life or death. We can worry about muh policies after the power structure of the enemy is annihilated.”
Topic 4 Ideology	Terms that relate to the ideology (e.g., fascist, socialist, nationalist) and beliefs (e.g., party, system, govern) of right-wing extremist groups	Nation, politics, social, people, world, movement, party, war, hitler, govern, power, system, german, socialist, American, European, nationalist, country, fascist, economy, ideology, history	Intragroup Debate	“National Socialism is believing in the People’s Community and taking from the Right nationalism, without capitalism and from the Left, socialism without internationalism”

Using the average cosine similarity on the normalized pointwise mutual information values, we identified that the three-, six- and 15-topic models had the highest statistical coherence scores (Röder et al., 2015; Figure 1). We therefore retained the three-, six-, and 15- topic models for the analyses in Part 3, alongside the four-topic model. We gave each of the topics a representative label, based on the terms included in the topic (Table 3; Supplementary Tables 4–6).

### Discussion

The topic modeling replicated and extended the findings of Part 1 across the whole data set by demonstrating that the qualitatively coded categories could be mapped at scale. Moreover, the topic modeling provided a method by which to quantify the content of the posts so that we could use the content (probability distributions of the topics within each post) as features (similar to variables or predictors) in the machine learning models in Part 3.

## Part 3: Machine Learning Modeling

Our final analyses addressed the primary empirical aim of our research: to investigate whether digital traces can be used to identify signals of mobilization to extremist action. Our sample of extremists who had and had not mobilized enabled us to do this, as we could use mobilization status as a binary target variable, with the aim of classifying whether a post was authored by a user who had mobilized or not. Therefore, in Part 3, we developed and tested random forest classifiers, which used features (similar to variables or predictors) created from the topic distributions in the topic models tested in Part 2, in addition to paralinguistic features and metadata (see Scrivens et al., 2023). We included paralinguistic features in the modeling as they can represent what could be considered leakage of motivation to act and can signal intensity or “emotive force” (McArthur et al., 2018; Quirk et al., 1985; Rubin & Greene, 1992; Scrivens et al., 2023).

### Method

#### Sample

Our sample of posts for the analysis in Part 3 was the same as that used in Part 2. However, prior to our analysis we needed to derive the features that would be used in our machine learning models. First, drawing on the topic models identified in Part 2, we extracted the probabilities of each topic occurrence per post (i.e., for the four-topic model, each post had four probabilities, one for each topic). This provides a set of topic distribution scores for each post (for example, a post might consist of 10% Online Communication, 40% Hate Speech, 40% Violent Action, and 10% Ideology) that could be used as features in our machine learning modeling.

Next, we considered alternative variables beyond thematic textual content that might inform differences between individuals who had mobilized and individuals who had not, that could be used as additional features in our models. We then generated paralinguistic features and word count data from the posts, drawing on evidence suggesting that these can be used to distinguish between online users (Golder & Donath, 2004; Scrivens, 2023). Specifically our features included (a) word count data using tokenization on whitespaces (Horne et al., 2017), (b) the number of links shared in a post based on the use of “http” and “www” (Hine et al., 2017), (c) question marks (Bhatia et al., 2016; Nguyen et al., 2022), (d) exclamation marks (Nguyen et al., 2022), and (e) total punctuation used (Horne et al., 2017; Mihaylov & Nakov, 2019). All counts of punctuation in posts excluded the punctuation included within links and hyperlinks.

Finally, due to sparsity of information in some posts and variance in length, we summarized users’ posts by averaging the feature values of randomly selected subsets of 10 posts. This provided the model with more task-relevant information and helped ensure the model was learning relevant statistical constructs, leading to improved informed decisions, while keeping the number of observations as high as possible. This left us with *n* = 11,979 observations (with 8,705 from nonmobilized users and 3,274 from mobilized users) for our machine learning models.

#### Analytic Strategy

For our modeling, our classification target was mobilization status—a binary variable representing whether an individual had mobilized or not. Due to their typically high performance on tabular data sets out-of-the-box, we opted for random forests^4^ as our choice of algorithm for this task (Breiman, 2001; Wright & Ziegler, 2017). We deployed the “ranger” random forest implementation in R (Wright & Ziegler, 2017) via the *mlr3* (Lang et al., 2019) framework. In total, we ran nine specifications. The first four used the topic distribution probabilities for each post as features in our machine learning task for the three-, four-, six- and 15-topic models separately (see Table 4; Supplementary Table 7). The next four included the same topic distributions as features, in addition to paralinguistic features (i.e., number of web links, exclamation points, and a count of punctuation) and word count (Nguyen et al., 2022; Waseleski, 2006). The final specification only included paralinguistic features and word count (see Supplementary Table 8). This latter specification enabled us to investigate the performance drop when only including topic distributions in the models and confirm the topic distribution features were contributing to the accuracy of the model.

**Table 4.** Results of Random Forest Models Predicting Mobilization to Extremist Action (Mobilized Sample n Posts = 3,274, N Users = 26; Sample Who Had Not Mobilized n Posts = 11,979, N Users = 48).

Evaluation metric	3 topics, paralinguistic features/word count	4 topics, paralinguistic features/word count	6 topics, paralinguistic features/word count	15 topics, paralinguistic features/word count	3 topics	4 topics	6 topics	15 topics
Accuracy	0.756	0.767	0.762	**0.768**	0.714	0.734	0.742	0.757
AUC	0.709	**0.713**	0.706	0.710	0.573	0.580	0.588	0.606
Balanced accuracy	0.630	0.635	0.634	**0.637**	0.507	0.509	0.516	0.521
F1 score	0.414	0.405	0.410	**0.428**	0.127	0.120	0.124	0.115

*Note*. Bold text denotes best performance; underlined text denotes second best performance.

All models were evaluated with a fivefold cross-validation (CV) and 100 trees, which we found to be a good balance between under- and overfitting. Moreover, in our CV, we employed blocking on the user ID to ensure that posts from the same user did not appear across multiple folds, which would cause a data leakage between train and test set (where models could learn specific user behavior instead of conviction status and artificially inflate performance). We decided against a higher number of folds in the CV, as this led to instabilities in the label distribution (e.g., only users who had mobilized in onefold) due to the user ID blocking.

We did not conduct further hyperparameter tuning on the random forests (besides testing different numbers of trees, which provided similar results), as tuning, especially with that many different specifications, becomes computationally expensive in terms of power and time due to it requiring a nested evaluation (with both inner- and outer-sampling for tuning and testing). Moreover, using a nested evaluation while ensuring no data leakage combined with a stable label distribution across folds would be almost impossible to set up due to our limited sample size. Therefore, we decided to use random forests instead of other classifiers, as even with little to no tuning they generally obtain excellent out-of-sample performances (Borisov et al., 2022; Fernandez-Delgado et al., 2014). To evaluate our models, we used area under the curve (or AUC; measuring how well the model can distinguish between two classes), overall model accuracy, balanced accuracy (how well the model classifies posts for those who had mobilized and those who had not), precision (the accuracy of the positive predictions), recall (the completeness of the positive predictions), and the F1 score (the harmonic mean between precision and recall).

Furthermore, to aid interpretation of the features and their relationship with conviction status, we report feature importance and Shapley values for the best performing specifications. The permutation feature importance scores for each feature are derived by permuting the respective feature (i.e., for each observation randomly swapping the feature value with that of another observation) and evaluating by how much the classification error (on the out-of-bag data sample for each tree) increases on average (over all observations). We derived separate scores for each of the models produced by the fivefold CV, which we subsequently averaged across the folds to obtain our final importance scores. Thus, for each feature, the importance score indicates how much the classification error would increase if we removed its relationship to the target variable. These scores come with the caveat that because features work together in a classifier to predict the outcome (and are not treated as independent predictors), features might have interactions between them which may lead to slight overestimation of the importance of a single feature, or they might be correlated and therefore affect the outcome via the same channel (for example, word count and more punctuation are both likely to be positively related to the length of a text, and if the longer text is the main predictor of the target, the importance will be distributed somewhat equally between both features). Finally, to understand the direction of the effect of each feature on the outcomes (mobilized vs. had not mobilized), we computed Shapley values. These were obtained through the *TreeSHAP* algorithm implemented in the R package “treeshap.” We derived the Shapley values for each of our test observations for the first model in the fivefold CV only, as averaging or summarizing them across all five models is not possible.

### Results

The results of our random forest modeling are in Table 4 and Supplementary Tables 7 and 8. We found that the specification that included the four-topic distributions (informed by the results of the content analysis) plus paralinguistic features and word count had the best performance in terms of AUC (independent of the exact classification threshold), followed by the 15-topic model, plus paralinguistic features. In most of the other metrics (Accuracy, Balanced Accuracy, F1 score; sensitive to the exact classification threshold), the two specifications also performed best. We, therefore, retained these two specifications for computing permutation feature importance and Shapley Values.

**Figure 2.**
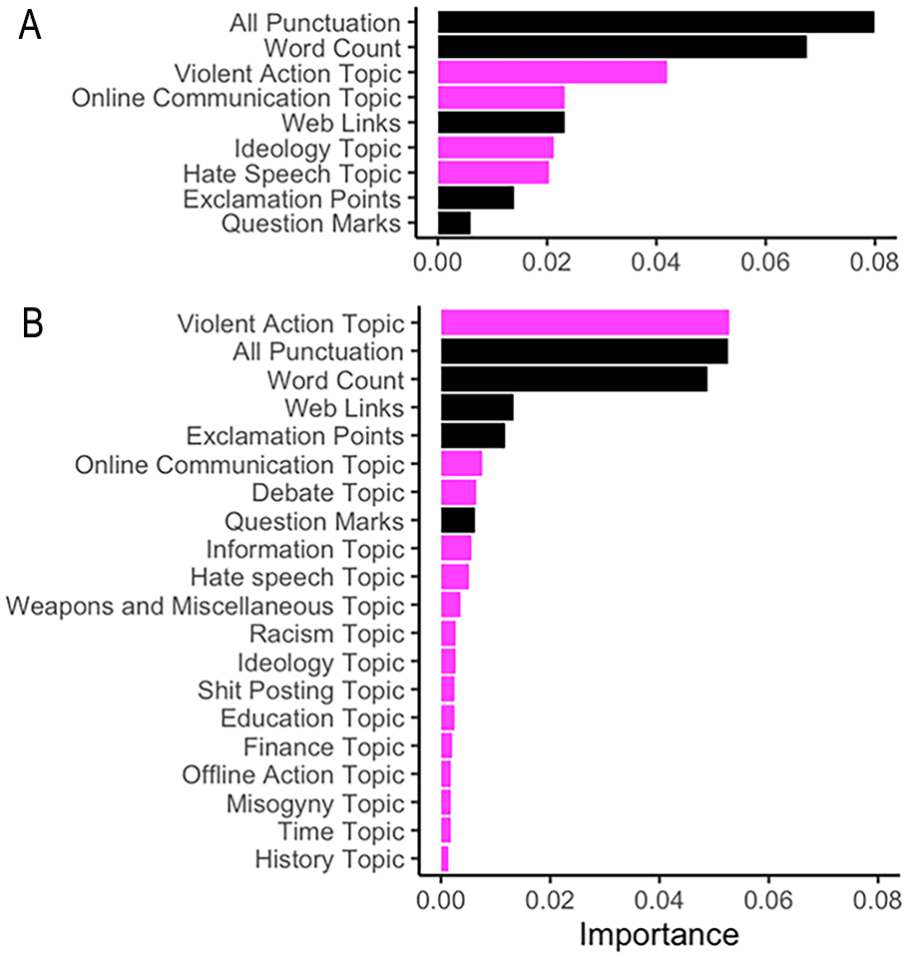
Permutation Feature Importance for the Topic (Magenta) and Paralinguistic (Black) Random Forest Model Specifications: (A) Four-Topic and Metadata Features and (B) 15-Topic and Paralinguistic Features.

Permutation feature importance scores for both specifications are visualized in Figure 2. The paralinguistic and word count features showed consistent importance in distinguishing between individuals who had mobilized and those who had not, with the features for punctuation and word count having the highest importance. In relation to textual content, the Violent Action feature in both the four- and 15-topic specification was most important in distinguishing between individuals who had mobilized and those who had not (Figure 2). The terms within the Violent Action topic for both the four- and 15- topic included “terrorist,” “gun,” “attack,” “punch,” and “police” (see Table 3 and Supplementary Table 6). Notably, in the specification that included the 15-topic distributions, Violent Action outperformed the paralinguistic features and word count, and it was the most important feature overall in distinguishing those who had mobilized and those who had not (Figure 2B). The Ideology and Hate Speech (the specifications that four-topic model plus additional features) and topics relating to Historical Discussion, Time, and Misogyny (15-topic model plus additional features) were found to contribute the least to the model.

**Figure 3.**
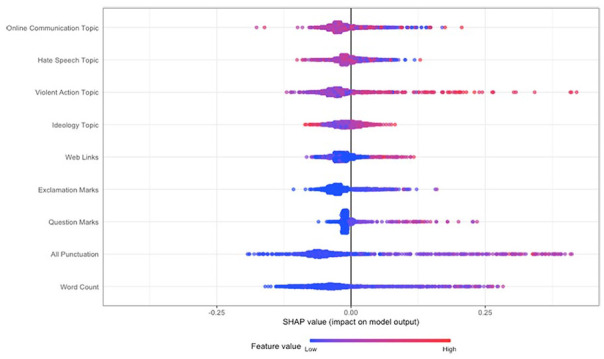
Shapley Values for the Random Forest Model Specification With Four Topics and Paralinguistic Features/Word Count.

**Figure 4.**
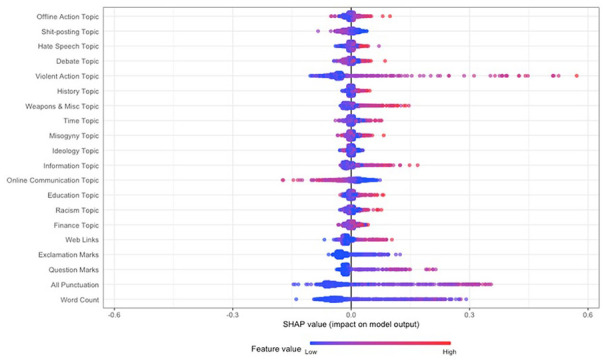
Shapley Values for the Random Forest Model Specification Including 15 Topics and Paralinguistic Features/Word Count.

The feature importance scores show the order of importance of features in the classification task, but not the direction of the relationship between the classes and the features (i.e., whether a feature is likely to predict a post being authored by an individual who had mobilized or not). Therefore, to gain insight into the relationship between the features and the classes, we computed Shapley values using TreeSHAP (Lundberg et al., 2020; Figures 3 and 4). Shapley values to the right of the plot contributed positively to classifying posts as being authored by a user who had mobilized to extremist action (increase in predicted probability for mobilization as indicated by the x-axis), while values to the left contributed negatively (decrease in predicted probability). A red point denotes that the feature of the respective observation that the Shapley value was computed for was higher (e.g., Violent Action, higher word count), while blue denotes a lower feature value (e.g., lower word count).

Based on the specification that included the four-topic distributions and paralinguistic features (i.e., the topic model that aligned with content analysis; Figure 3), we observed that increases in discussion of the Violent Action topic, increases in punctuation, and a higher word count (demonstrated by the red becoming more prominent) increased the predicted probability that an observation was authored by an individual who had mobilized (falling to the right of the plot, with positive Shapley Values). Furthermore, the topic of Violent Action (and punctuation) had a greater impact on the model predictions (demonstrated by the long tail to the right). In contrast, increases in the Ideology and Hate Speech topic decreased the predicted probability that an observation was authored by an individual who had mobilized (in red, falling to the left of the plot, with negative Shapley values), hence were more indicative of posts from users who had not mobilized. The same result was found for less punctuation and a lower word count (denoted by more prominent blue).

The Shapley values for the specification containing the 15-topic distributions and paralinguistic/word count features (Figure 4) replicated the previous result, suggesting that increases in the Violent Action topic were associated with an increase in the probability that the observation was authored by an individual who had mobilized (denoted by increasingly prominent red) and had the largest impact on (some) model predictions (denoted by the long tail on the right). Increases in the topic features relating to Weapons and Miscellaneous, Misogyny, Information, question marks, and punctuation were related to an increase in probability of the post being authored by an individual who had mobilized. In contrast, both increases in the Online Communication topic and decreases in the Violent Action topic decreased the probability of a post being authored by an individual who had mobilized.

### Discussion

In Part 3, we investigated whether digital data could be used to identify signals of mobilization to extremist action by building a series of random forest models. Across the best performing models, we found that Violent Action content, in addition to punctuation and word count, was the most important feature in distinguishing between those who had mobilized and those who had not. Significantly, Violent Action terms had a higher probability of occurring in posts authored by mobilized users, as did increased punctuation and length of post. Taken together, these findings provide evidence of leakage in the online posts of individuals who have mobilized to extremist action (Meloy & O’Toole, 2011). Specifically, and informed by our qualitative analysis in Part 1, we suggest that the Violent Action terms are indicative of leakage of capability and a focus on action—that is they make explicit references to the resources and behaviors required to undertake extremist action (Boyns & Ballard, 2004).

In contrast, our findings for both the four- and 15-topic models suggest that hate speech and ideological content reduced the likelihood of a post being authored by an individual who had mobilized. This is at odds with the long-standing focus on ideology and hate speech in terrorism studies and grievances and anger in collective action research (Ackerman & Burnham, 2021; Holbrook & Horgan, 2019; van Zomeren et al., 2008). The finding suggests that the expression of the motivation for action (e.g., ideology, anger) is not sufficient to indicate that future action is likely and therefore cannot inform our understanding of the psychological tipping point to action (see Livingstone, 2014). Importantly, it strengthens the need to distinguish between identifying beliefs and motivations for extremist action versus the knowledge and behaviors that both enable and indicate that mobilization is likely to take place (Borum, 2011; Khalil, 2014).

## General Discussion

The purpose of this research was to investigate the digital indicators of extremist mobilization. Our findings provide insight into the psychology of mobilizing, as well as how people who later mobilize behave online. In turn, the results can be used to help solve the specificity problem (Sageman, 2011). By using data from people who expressed extremist beliefs but did not mobilize as a baseline comparison group, our approach has enabled us to show that expressing ideological views is not necessarily part of the mobilization process, at least in that expressing those views online does not differentiate mobilization from nonmobilization. As such, expressing extremist views in and of itself does not signal that the individual will mobilize to offline extremist collective action (there are, of course, other potential harms in that ideological content can mobilize other individuals to act, Borum, 2011). Instead, we found that future offline mobilization is associated with talk of violent action, capability, and know-how, alongside emotional intensity.

There are three possible interpretations for these findings regarding the violent action topic. First, talk of violent action may suggest that people are using online interactions to acquire know-how to increase their capability to act. Second, such talk may be evidence of leakage of the intention to act or of the planning of action; and third, talk of violent action may be more common in the mobilized sample because they were more focused on action, weapons, and so on. As this work is exploratory and data-driven, we cannot be certain which of these interpretations is most apt. However, the first interpretation aligns most with the results of our content analysis in Part 1, which was designed to illuminate and add richness to the topic modeling. The content analysis showed that when we consider the use of the violent action terms within sentences, the first interpretation is most accurate: Individuals appear to be equipping themselves with knowledge about weapons, avoiding detection from law enforcement, and preparing to engage in offline actions. This is consistent with prior case study–based research, which identified that 40 of 49 convicted terrorists had used the internet to acquire training and information to plan their attacks (Gill et al., 2017) and aligns with research on the indicators used by law enforcement, which found that evidence of an individual engaging in surveillance and observation were important factors when identifying the threat of terrorism (Gruenewald et al., 2018).

### Conceptual Contributions

Our findings add to the understanding of how users of online platforms who are active participants in the planning and execution of extremist action offline might behave differently to consumers/sharers of extremist content (Gill et al., 2017; Schuurman et al., 2018). In line with resource mobilization theory (Ferree & Miller, 1985; McCarthy & Zald, 1977), the results suggest that it is not the discussion of ideology or the expression of hateful views that are associated with subsequent mobilization (indeed these decreased the probability that the user mobilized), but references to violence, weapons, and the types of behaviors and actions that will enable them to perform more effectively (i.e., logistics planning, avoiding detection by law enforcement). This finding supports suggestions that theories of mobilization and radicalization have, at present, underplayed the importance of resources and capability-focused behaviors when determining the psychological “tipping-points” for an individual to act (Beck, 2008). Indeed, while theories of radicalization (McCauley & Moskalenko, 2017) and mobilization (van Zomeren et al., 2018) tend to focus on the collectivization of efficacy beliefs (in terms of the efficacy of the actions rather than the actors), and expression of grievances, the implication of our finding is that, to be able to account for the initiation of action (“activation”), they should (also) account for the development of individual actors’ *self*-efficacy, understood as the development of know-how, resources, and preparedness to act (rather than belief in the political efficacy of the collective action, as per van Zomeren et al., 2008). This suggests that the radicalization and collective action literature may benefit from integration of ideas on capability or self-efficacy development from (for example) resource mobilization theory (Beck, 2008) and the theory of planned behavior (Ajzen, 1985; Rottweiler & Gill, 2022).

Alongside evidence of leakage of intention to mobilize in the content of posts, we also found that the online posts of users who mobilized (relative to those who did not) were longer and contained higher levels of punctuation. This may indicate an intention to “exclaim”: express greater intensity of feeling (McArthur et al., 2018; Quirk et al., 1985; Rubin & Greene, 1992). This resonates with evidence that approach-oriented emotions such as anger and moral outrage are predictors of the motivation for collective action (Spears et al., 2011; van Zomeren et al., 2004, 2008). People in hyperpartisan online communities are more likely to use punctuation such as exclamation points—perhaps in an effort to try to engage others (Nguyen et al., 2022). Our results suggest that this emotional intensity may “leak” through online posts, and the difference between classes suggested that this signal was stronger for people who mobilized than for those who may believe in the justification for collective action but did not intend to mobilize themselves.

Our second major finding was related to hate speech and ideological content. While the qualitative analysis suggested there was no difference between the two groups in the extent to which they engaged in debate about extremist ideology, the quantitative modeling showed that hate speech and ideological content reduced the likelihood that a post was authored by a user who mobilized. Therefore, this extremist online behavior was not, in and of itself, positively related to the risk of extremist mobilization, and focusing on ideology in algorithms may actually undermine efforts to detect persons of interest (Mussiraliyeva et al., 2020; Scrivens et al., 2020; Zannettou et al., 2018). This contrasts with the focus on ideology in research that aims to understand the perpetration of terrorism and political violence (Ackerman & Burnham, 2021; Holbrook & Horgan, 2019; Scrivens et al., 2018; Webber & Kruglanski, 2018). We do not dispute that ideological beliefs can motivate an individual to perpetrate an actor of terror (Webber et al., 2020); however, our results indicate that the expression of these beliefs does not provide a sufficient signal to indicate that future action is likely. As such, while it remains useful to analyze hate speech and ideological content to understand why an individual might identify with a specific extremist group and begin expressing views that align with that of the group (i.e., to understand radicalization processes; Lee & Knott, 2022), our findings support Sageman’s assertion that “many people say very violent things, but very few follow up with violent actions” and bolster Borum’s argument that greater consideration is needed in separating extremist ideology from extremist action (Borum, 2011; Sageman, 2011).

### Practical and Ethical Implications

We provide preliminary evidence of online behaviors that might indicate or “leak” mobilization to extremist action (consistent with prior research on case studies, Dudenhoefer et al., 2021). These digital “risk signals” of extremist mobilization could potentially be identified within users’ social media data and therefore facilitate practitioners in prioritizing resources in the monitoring of online material, even in instances where the authors behind posts are not known. At the same time, there are ethical sensitivities and dual use concerns associated with this. Indeed, while all the data we collected were in the public domain and accessible via public repositories, ethical considerations remain on the use of social media data where users express viewpoints that include hate speech or incitement of violence. If our machine learning algorithm is misapplied and not used for data triage purposes, in conjunction with additional data and investigations, the algorithm could be misused to identify persons of concern. As no algorithm is ever 100% accurate, this application would be inappropriate. To avoid our findings being misapplied and/or misused, practitioners should see these insights as one of several ways to triage the large volumes of data they encounter. These concerns should also be weighed against the drawbacks of not sharing the findings, which could improve public safety, and inform future work on extremist mobilization—particularly in terms of digital traces.

### Limitations and Future Directions

As with all research seeking to link digital data to offline outcomes, our findings are not without limitations. First, it is important to note that our findings only apply to individuals who have used social networking platforms prior to mobilization. While the majority of individuals identified in our database as being convicted of right-wing extremist offenses were known to be users of social networking platforms, not all of them were. Thus, it is important that other (i.e., nondigital) indicators of future mobilization continue to be examined. Second, it is not possible to determine whether our findings will apply beyond the context of extreme right-wing mobilization, and indeed we acknowledge the importance of cultural and language-specific indicators of extremism (Cohen et al., 2014). That said, a key contribution of our research is the methodological approach. The qualitative analysis provides a nuanced and fine-grained understanding of the data being examined and, crucially, accounts for the context in which individuals are communicating. Future research might apply our approach to the study of other extremist groups and further advance research seeking to understand the relationship between online and offline collective action (Greijdanus et al., 2020; Thomas et al., 2022).

We also acknowledge that machine learning models will never be wholly accurate in their predictions of human behavior and therefore suggest caution in the application of our findings. The data used in our models represent a snapshot of an individual’s online behaviors during the time that their data were obtained, and as such, can only provide a “cloudy window” of the signals of mobilization (Smith et al., 2023). There are, of course, a number of other important indicators that might be used to determine the likelihood of extremist mobilization (e.g., criminal history, Shortland et al., 2022). Accordingly, future research might focus on triangulating data from a wider range of sources.

Data availability, as well as unobservable behavioral signals, will always remain a challenge. Our approach attempts to overcome some of these difficulties by employing a novel ground truth data set that enables us to compare posts and online behaviors across groups. There are, however, limitations associated with our sample, not least due to its size. Some people in the sample of users who had not mobilized to extremist violence at the time of the research may do so in future (but at the same time their online behavior may change when they decide to initiate action). The timescales from authoring posts to mobilizing to extremist action also varied within our sample, as well as the types of action in which they engaged. These variations could, of course, have influenced the findings such that posts authored by individuals who committed certain types of offenses (e.g., violent) or who posted a higher proportion of posts in the time period immediately preceding their offense, might have disproportionately contributed to the accuracy of our models. While the variation, particularly in terms of the timing of posts, limits what we can conclude about when behaviors might occur prior to mobilization, it also means that our predictors of mobilization are not, in fact, just evidence of mobilization itself, but are behaviors enacted *prior to* mobilization (and that therefore provide an a priori signal thereof).

Furthermore, given the scarcity of ground truth data in this field and the difficulty of obtaining the type of empirical data (see Khalil, 2014), we suggest our analyses and data set provide initial novel insights into the mobilization process, which can then be extended in future research and perhaps combined with additional data sets (see Giner-Sorolla et al., 2024). For example, future research might focus specifically on posting trajectories (of both content and frequency of posts) to determine whether there are other patterns of behavior that are associated with future mobilization (see, for example, Scrivens et al., 2020).

Finally, as we explained above, there are three potential explanations for our finding that the users who mobilized engaged in more talk of violent action than those who did not, and as this work is exploratory, we cannot be certain which of these interpretations is most apt. Therefore, future research should investigate explanations for the relationship between talk of violent action, including discussions that might enhance capability and know-how, on mobilization. Such research is likely to require additional ground truth data that indicate which of our proffered explanations is most likely and might involve a theoretically informed examination of the use of the internet in convicted terrorist offenders (see Kenyon et al., 2022) and, if possible, interview data.

### Conclusion

In conclusion, we suggest that understanding extremist mobilization and the initiation of action requires different theorizing than radicalization (or support for extremist collective action)—as they involve different processes (or at least different phases of a process). Our findings suggest that people who are intent on mobilizing to extremist action are likely to post content about violent actions, operational planning, and logistics, as well as “leaking” emotional intensity through paralinguistic cues. In contrast, both people who support extremist action but are not intent on mobilizing, and people who are, post ideological and hateful content, so this content cannot help elucidate the mobilization process. To enable accurate explanation and prediction of mobilization, theories of collective action and mobilization need to describe the behaviors and conditions that lead to a radicalized individual passing a psychological tipping point that enables action. In turn, these insights and methods may help law enforcement personnel to identify the “needles” of terrorism in an ever-growing “haystack” of extremist content.

## Supplemental Material

sj-docx-1-psp-10.1177_01461672241266866 – Supplemental material for Online Signals of Extremist Mobilization Supplemental material, sj-docx-1-psp-10.1177_01461672241266866 for Online Signals of Extremist Mobilization by Olivia Brown, Laura G. E. Smith, Brittany I. Davidson, Daniel Racek and Adam Joinson in Personality and Social Psychology Bulletin

## Notes

1. We define extremism (and relatedly extremist content) as expressions of a belief system based on the hostile actions of an ingroup toward a perceived outgroup, which may or may not involve terrorist acts and in which that hostile action must be unconditional and tied to the ingroup’s perceived success or survival (Berger, 2018; Bötticher, 2017).

2. We define right-wing extremism as “a broad range of ideologies that essentially see violence as a legitimate tool to combat a political and ethnic ‘enemy’ (including individuals with different culture, religion, nationality or sexual orientation) seen as a threat to the own race or nation” (Koehler, 2014: 51).

3. The conviction status of users is correct up until the point that the paper has been submitted for publication. We recognize that there is a possibility that individuals included in our nonconvicted sample could commit a terrorism-related offense at a later date.

4. For further information on random forests and machine learning methods in psychological research, please see Fife and D’Onofrio (2023) and Tay et al. (2022).
