# Supplementary material for: Online Signals of Extremist Mobilization
Source: Pers Soc Psychol Bull. 2024 Jul 31;52(1):70–89. doi: 10.1177/01461672241266866 (PMC12681366; doi:10.1177/01461672241266866)
Supplement: sj-docx-1-psp-10.1177_01461672241266866 – Supplemental material for Online Signals of Extremist Mobilization [file sj-docx-1-psp-10.1177_01461672241266866.docx]

**Supplementary materials**

**Contents**

Table 1 ………………………………………………………………………………………….2

Table 2………………………………………………………………………………………….3

Table 3………………………………………………………………………………………….5

Table 4……………………………………………………………………………………….…11

Table 5………………………………………………………………………………………….12

Table 6………………………………………………………………………………………….13

Table 7………………………………………………………………………………………….15

Table 8………………………………………………………………………………………….16

**Table 1**

*Coding framework to match digital aliases (i.e., usernames) to the identity of the users within our dataset*

| *Feature* | | *Weight of feature* |
| --- | --- | --- |
| Clear identifying reference to own identity on page/chat log  (e.g., Gab page with real name) | | 3 |
|  |  |  |
| Multiple credible online sources demonstrating username-identity match (e.g., court records, news reports) | | 3 |
|  |  |  |
| Identifying information  (e.g., reference to known workplace, own name, declaration of their position within an organization) | | 2 |
|  |  |  |
| Photographic evidence included in the chat log/profile  (e.g., posted photo of self in chat) | | 2 |
|  |  |  |
| Reference to known geographic location of individual in text  (e.g., reference to attending local gathering of other extremists) | | 1 |

*Note.* Features are given a weighting of 1-3. To be confident of a username-identity match, a score of 5 had to be obtained.

**Table 2**

*Inclusion criteria for non-convicted right-wing extremist sample*

| *Feature* | *Weight* |
| --- | --- |
| Publicly known figure in the far-right(e.g., their own Wikipedia page, owner of far-right website)  (Jensen & LaFree, 2016) | 8 |
|  |  |
| Far-right referenced in username (e.g., Hitler, Aryan, Hauptsturmfhurer)  (Holt et al., 2018; Jaki & De Smedt, 2019; Smith, Wakeford, et al., 2020) | 2 |
|  |  |
| Far-right referenced in avatar (e.g., pepe the frog, swastika)  (J. M. Berger & Morgan, 2015; Holt et al., 2018; Smith, Wakeford, et al., 2020) | 2 |
| Racist content (e.g., hateful and extremist language)  (Bowman-Grieve, 2009; Scrivens, 2021b; Zannettou, Bradlyn, De Cristofaro, Kwak, Sirivianos, Stringini, et al., 2018) | 2 |
| Portray self as far-right in messages (e.g., references self as part of a known organization, “based”, “red-pilled”)  (Munn, 2019) | 2 |
| Reference to other well-known perpetrators in far-right (e.g., Dylan Roof, Timothy McVeigh, Robert Bowers)  (Conway et al., 2019) | 2 |
| Homophobic content  (Bowman-Grieve, 2009; Jaki & De Smedt, 2019; Scrivens et al., 2020) | 1 |
| Sexist content  (Jaki & De Smedt, 2019) | 1 |

*Note.* Features are given a weighting of 1-8. To be confident of user being identified as a right-wing extremist, a score of 8 had to be obtained.

**Table 3**

*Coding protocol for content analysis*

| Higher-order category | Sub-category | Description | Example |
| --- | --- | --- | --- |
| Group Formation | Recruitment | Forming a group and connecting other forum users together | “ Anyone wanting to get involved with [redacted] in London then email [redacted] or via the  contact tab on the regional blog” |
|  | Connecting with others |  | “ You guys know of any missouri guys here?” |
|  | Transnational connections |  | “Hi mate, I'm one of the main organisers with [redacted] in the UK. Who is in charge of [redacted] at the moment? I want to establish contact. Thanks” |
|  | Organizing and setting up |  | ““@redacted I suggested that my chapter operate in such a way that we could get our chapter objectives done via different groups (or 'cells') throughout my area [redacted]” |
| Hateful Content | Hatespeech | Hateful and derogatory content in relation to extreme right-wing beliefs – racism, homophobia, misogynism etc. | “6 words speech. Gas the kikes. Race war now” |
|  | Glorifying XRW violence |  | ““All hail Robert "Real White Power Hours" Bowers! #HeroRobertBowers #SaintRoofWouldBeProud #HeroWeNeedButDontDeserve” |
|  | Shitposting |  | “African gas chambers would be sticking you in a hut with dead animals until you died” |
| Information Sharing | Sharing literature | Information being shared to other members of the online forum | “ A massive nazi archive with every speech, documentry, book, ect on the subject, lots of military history. Also includes a collection of fine classical music and essays on philosophy.  [LINK]” |
|  | Sharing news |  | “[LINK]  [LINK]  Stop immigration - start repatriation.” |
|  | Sharing an event |  | “Less than a week until the start of my college tour at the University of [redacted] on Saturday the 17th. If you haven't yet registered, there is still time! Sign up at [LINK]” |
|  | Creating and sharing propaganda |  | “I combined old NSLF propaganda with [redacted]. Here's a few pieces I made:” |
| Intragroup Debate | Strategizing | Debate amongst members of the forum, including ideological debate, historical debate and debate around group norms | “ To achieve the 14 Words, we need our own sovereign territory where our People can not only survive but thrive. We can not take it all back so breaking off a piece proportional to our strength & capacity to do so is the next best option.” |
|  | Historical Discussion |  | “Was cold war france really a free market american puppet? You should be thanking your lucky stars france did not go red, If you don't like trotskyites that is. 1930's certainly all the sorelian and right wing socialists all went off to form fascist movements leaving only absurd people” |
|  | Debating group norms/ membership |  | “Ok first of all, no homosexuals are allowed in our Party. Second of all, if someone was into something degenerate before becoming a National Socialist and then National Socialism had them change their life, what's the issue?” |
|  | Debating philosophy/ideology |  | “ National Socialism believes in the People's Community and taking from the Right nationalism without capitalism and from the Left, socialism without internationalism” |
|  | Debating current events |  | “They are clearly positioning for Syrian interventionalism based on this gas attack from the other day which seems to be entirely faked/staged bullshit” |
| Operational | Operational and security | Advice on how to operate effectively | “We've had to rethink some of what we were doing based on intel and seeing the rally last weekend.” |
|  | References to law enforcement |  | “you do realise that [redacted] are involved with this right?” |
|  | Doxing |  | “If you're afraid of being doxxed you probably should not go. That being said, we need to stop being afraid of being doxxed.” |
| Threat of Violence | - | Content that reflects an individual threatening to engage in act of violence. | “Whites *did* have privilege but they squandered it & now we're here. We need to police our own Racial ranks first. Join us or stay out of our way, otherwise you are the enemy.” |
| Offline Action | - | Reference to action offline – flyering, postering, lobbying etc. | “Went leafleting for a nationalist party in the UK today - the response was heartening, I will be out again tomorrow. We march forward” |
| Weapons | - | Mention of weapons | “ my glock family. with a plastic training and kkm 40s&w barrels for each. truglo tfx on the 31 and xs big dots on the 32&33. and grip tape on the gen 3 on the right so it's not so greasy when wet. edit: in this pic 33 still had tfx sights” |
| Inciting Violence | - | Content that incites other people to engage in acts of violence | “The cost of one bomb is equal to 60 million leaflets (which the Movement could neither produce nor distribute). Terrorism is good economics...The public is at once informed & this information could not have been spread more cheaply or more quickly." |

*Note.* Identifying information has been removed and some quotations have been paraphrased to protect the anonymity of the individuals in our dataset (see Brown et al., 2022; British Psychological Society, 2021).

**Table 4**

*Topic model with 3 topics in mobilized (n posts = 32,619, N users = 26) and users who had not mobilized (n posts =86,854, N users = 48)*

| Topic | Example Terms |
| --- | --- |
| V1  Violent Action | Antifa, Charlottesville, terrorist, ni*ger, gun, attack, cville, rally, police, look, wear, arrest, shirt, police, shoot, mask, night, punch |
| V2  Online communication | Post, shit, gab, people, account, jew, time, stormer, kike, everyone, talk, tell, site, ban, server, meme, internet, follow, link, discord |
| V3  Ideology | Nation, white, people, war, race, world, country, american, social, american, jew, hitler, christian, nationalist, movement, christian, german, fight, europe |

*Note.* “cville” = Charlottesville, “stormer” = Daily Stormer (alt-right news platform).

**Table 5**

*Topic model with 6 topics in mobilized (n posts = 32,619, N users = 26) and users who had not mobilized (n posts =86,854, N users = 48)*

| Topic | Example Terms |
| --- | --- |
| V1  Politics | White, jew, trump, people, race, vote, speech, jewish, racist, Israel, support, America, democrat, conservative, media, antiwhite, president, elect, control, marriage, law |
| V2  Ideology | Nation, war, German, world, Hitler, European, socialist, Christian, fascist, political, british, economy, power, history, revolution |
| V3  Violent Action | Antifa, Charlottesville, terrorist, attack, cville, rally, police, violent, arrest, wear, cop, mask, defence, protest, gun, thus, torch, charge |
| V4  Online Communication | Post, read, server, stormer, account, chat, gab, link, email, everyone, discord, siege, channel, site, check, pm, welcome, send, video, episode, vet, join |
|  |  |
| V5  Hate speech | Fuck, nig*er, shit, fa**t, ki*e, gay, kid, ass, girl, dude, whore, dick, women, bitch, fat, ni**a, sex, rape, kill, woman, suck, jew, gas |
| V6  Debate | Something, people, person, actual, movement, serious idea, inform, reason, internet, active, obvious, debate, explain, communicate, discus, talk |

*Note.* “cville” = Charlottesville, “stormer” = Daily Stormer (alt-right news platform).

**Table 6**

*Topic model with 15 topics in mobilized (n posts = 32,619, N users = 26) and users* *who had not mobilized (n posts =86,854, N users = 48)*

| Topic | Example Terms | |
| --- | --- | --- |
| V1  Offline action | Flag, march, event, dawn, rally, red, golden, attend, black, command, park, twp, plan, city, anniversary, patriot, meet, police, uniform, wear, weekend | |
| V2  Shit-posting | Sieg, shit, fuck, gas, nig*a, dick, heil, ride, chamber, meth, lmao, stomp, kick, roll, cock, suck, smoke | |
| V3  Hate speech | Ki*e, jew, fuck, fa**t, ni*er, jwish, shit, retard, saint, stupid, idiot, bitch, bullshit, roof, | |
| V4  Debate | People, argument, understand, believe, something, reason, person, idea, explain, question, obvious, intellectual | |
| V5  Violent Action | Antifa, terrorist, attack, Charlottesville, cvill, violent, thug, defence, arrest, punch, suspect, cop, spray | |
| V6  History | Nazi, hitler, rockwell, enemy, jew, fight, evil, truth, George, Lincoln, hate, fascism, god, history, Nazism, communism, adolf, war, republic, neonazi, ancestor | |
| V7  Weapons and Miscellaneous | Radical, agenda, coffee, rang, song, shop, rifle, round, weapon, music, pistol, ammo, mag, | |
| V8  Time | Day, week, hear, minute, report, news, time, hour, ago, month, comment, radio, listen, late, morning, wait, hope, interview | |
| V9  Misogyny | Women, rape, girl, woman, ni**er, wife, sex, mother, love, marriage, kids, children, god, gang, parent, daughter, whore, gay, husband, rapist, slut | |
| V10  Ideology | Nation, European, socialist, Europe, govern, german, economy, war, Russia, capitalist, western, church, tradition, Marxism, Christian, society, | |
| V11  Information | Book, read, article, write, mein, written, mason, thread, relevation, club, forum, blog, website, material, recommend, inform, publish, pdf, translate, text | |
| V12  Online Communication | | Gab, speech, twitter, account, ban, post, server, free, chat, delete, discord, platform, facebook, censor, everyone, follow, tag, mail, |
| V13 Education | School, power, military, force, student, educate, system, arm, success, office, teaching, warfare, youth, prime, train, learn | |
| V14  Racism | White, race, vote, illegal, genocide, immigrant, supremacist, Africa, south, sharia, demographic, nonwhite, black, diversity, supremacist, ethnostate, racist, civic | |
| V15  Finance | Buy, money, donate, bitcoin, crypto, pay, fund, cash, print, dollar, fee, fundraise, mail, | |

**Table 7**

*Additional Results of Random Forest Models for Predicting Mobilization to Extremist Action (mobilized sample n posts = 3,274, N users = 26;*

*sample who had not mobilized n posts = 11,979, N users = 48)*

| Evaluation Metric | 3 topics,  para-linguistic features/word count | 4 topics, para-linguistic features/word count | 6 topics,  para-linguistic features/  word count | 15 topics, para-linguistic features/word count | 3 topics | 4 topics | 6 topics | 15 topics |
| --- | --- | --- | --- | --- | --- | --- | --- | --- |
| Precision | 0.548 | 0.520 | 0.532 | **0.611** | 0.181 | 0.218 | 0.237 | 0.297 |
| Recall | 0.333 | 0.331 | **0.334** | 0.330 | 0.098 | 0.083 | 0.084 | 0.071 |

**Table 8**

*Results of Random Forest Models for paralinguistic features only, Predicting Mobilization to Extremist Action (mobilized sample n posts = 3,274, N users = 26; sample who had not mobilized n posts = 11,979, N users = 48)*

| Evaluation Metric | Para-linguistic features/  word count only |
| --- | --- |
| Accuracy | 0.746 |
| AUC | 0.691 |
| Balanced Accuracy | 0.622 |
| F1 Score | 0.392 |
